# Casein kinase1α activators, a precision weapon for CRC

**DOI:** 10.18632/oncotarget.21892

**Published:** 2017-10-19

**Authors:** Bin Li, Ethan Lee, David J. Robbins

**Affiliations:** Bin Li: Molecular Oncology Program, Department of Surgery, Miller School of Medicine, University of Miami, Miami, FL, USA; Department of Pediatrics, Miller School of Medicine, University of Miami, Miami, FL, USA

**Keywords:** wnt, CK1α, phamacological activator, colorectal cancer, gastrointestinal toxicity

The evolutionarily conserved Wnt signaling pathway provides temporal and spatial cues during early embryonic development and during homeostatic function of numerous adult tissues [1]. Wnt signaling also drives the onset and progress of a broad array of human cancers, including nearly all nonhereditary colorectal cancers (CRCs) [1]. Despite the importance of this pathway in cancer, no Wnt inhibitors are currently clinically approved.

Wnt ligands are palmitoylated in the endoplasmic reticulum by the membrane bound O-acyltransferase, Porcupine, and palmitoylation of Wnt ligands is necessary for secretion of the active protein as well as high affinity binding to the Frizzled receptor [1]. To date, Porcupine inhibitors represent a particularly promising class of Wnt inhibitors that have proven effective in many Wnt ligand-driven preclinical cancer models. Porcupine inhibitors are currently being evaluated in clinical trials. Porcupine inhibitors, however, are unlikely to be useful for targeting CRC, the vast majority of which harbor activating mutations downstream of Wnt ligands. The critical event in the Wnt pathway involves post-translational regulation of the transcriptional co-activator β-Catenin. In the absence of Wnt signaling, β-Catenin is maintained at low levels in the cytoplasm via its association with a “destruction complex,” which consists of Glycogen Synthase Kinase 3 (GSK3), Casein Kinase 1α (CK1α), Adenomatous Polyposis Coli (APC), and the scaffold protein Axin. Within this complex, β-Catenin is phosphorylated, thereby targeting it for ubiquitin-mediated degradation [1]. The rate-limiting component in the destruction complex is Axin, and its steady-state protein levels are regulated by the poly-adenosine diphosphate-ribose polymerase, Tankyrase. Small-molecule Tankyrase inhibitors, which exhibited considerable efficacy in *APC* mutant CRC cell lines, have emerged as a promising class of novel therapeutics. However, Tankyrase inhibitors exhibit overt on-target gastrointestinal (GI) toxicity in mouse CRC models due to effects on the Wnt-dependent intestinal stem cells that maintain normal GI homeostasis [2]. Such a limited therapeutic index (at least in preclinical animal models) may ultimately limit progression of Tankyrases inhibitors into the clinic and their utility for CRC patients.

We initially described the identification of a mechanistically distinct Wnt inhibitor, pyrvinium, using a high-throughput small-molecule screen [3]. This first-in-class small-molecule Wnt inhibitor bound to and activated the protein kinase CK1α. Given the role CK1α plays as a negative regulator of Wnt signaling, this pharmacological activation of CK1α resulted in the reduced viability of CRC cell lines *in vitro*. Interestingly, pyrvinium was previously approved by the FDA and used successfully and safely for many decades in the clinic as an anti-pinworm medication. The bioavailability of pyrvinium was limited to the GI tract upon oral administration, a positive attribute for an anthelmintic drug, but one that limited its repurposing as a Wnt inhibitor for CRC patients. In a recent study, we described the development and characterization of a second-generation pharmacological activator of CK1α, SSTC3, which exhibited significantly improved bioavailability compared to pyrvinium [4]. Similar to pyrvinium, SSTC3 attenuated CRC growth *in vitro* and prolonged the survival of a mouse CRC tumor mouse model. Taking advantage of the improved pharmacokinetics of SSTC3, we showed for the first time the efficacy of CK1α activators in attenuating the growth of CRC *in vivo* using both cell lines and patient-derived CRC xenografts (including a lung metastatic cancer harboring a *KRas* mutation that was derived from a patient). We found that SSTC3 exhibited greater efficacy than a Tankyrase inhibitor in reducing CRC growth and demonstrated significantly less on-target GI toxicity in mice. We showed that this increased therapeutic index of SSTC3 was due to the differential abundance of CK1α, the direct target of SSTC3, in normal GI tissue relative to that in tumor tissue (Figure 1). We further showed that reduced levels of *CK1α* correlated with decreased survival of CRC patients [4]. These findings validate CK1α as a *bona fide,* druggable Wnt component in CRC and suggest that CK1α activators selectively target cancer cells. Consistent with our findings, CK1α activators have also been reported to exert efficacy as Wnt inhibitors in other disease models (e.g., myocardial infarction [5] and sarcoma [6]) without observable systematic toxicity.

**Figure 1:**
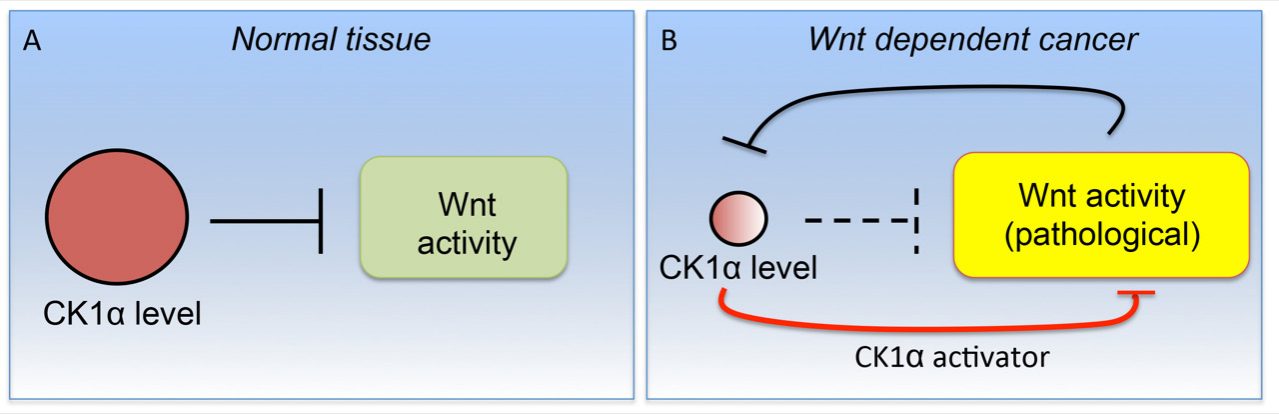
A schematic suggesting the mechanism underlying the enhanced therapeutic index observed with SSTC3 A. In normal tissue, constitutive CK1α levels are sufficient to maintain Wnt signaling at relatively low levels. B. In Wnt-dependent cancer cells, hyperactivated Wnt activity acts to suppress CK1α levels via an unknown mechanism. These reduced levels of CK1α result in its activity becoming rate limiting in Wnt-dependent tumors. In this context, pharmacological CK1α activators compensate for the insufficiency of CK1α activity, resulting in suppression of Wnt activity.

Despite the potential clinical advantage of CK1α activators, several mechanistic questions regarding their observed therapeutic index remain to be elucidated. One such question is understanding how CK1α pharmacological activators function mechanistically. While CK1 was originally proposed to be constitutively active, it is now clear that there are multiple CK1 family members and that their activities are highly regulated [7]. Our data indicate that CK1α activators bind to multiple CK1 family members but are only able to activate the intrinsic kinase activity of the CK1α isoform. Thus, it is likely that the binding of CK1α to agonists and its subsequent allosteric activation are distinct events, which may mimic an endogenous CK1α regulatory mechanism. Follow-up studies utilizing molecular modeling, mutagenesis, and structural analysis of CK1α bound to such pharmacological activators are therefore warranted in order to reveal the details of this apparent two-step process and to identify how this process is relevant to the regulation of endogenous CK1α.

A major question is how constitutive Wnt activity modulates CK1α abundance. There are many potential ways CK1α levels might be regulated in this context, a number of which have already been previously reported. At the genomic level, loss of regions of chromosome 5 (encompassing the *CK1α* gene) and methylation of the *CK1α* promoter have been observed. Non-coding RNA, microRNA-155, and specific transcription factors/co-factors (e.g., RUNX1) have also been reported to modulate *CK1α* expression. Finally, the stability of CK1α protein has recently been shown to be regulated by the chemokine SDF-1 as well as the E3 ligase CRL4(CRBN). Although these highlighted mechanisms have already been linked to Wnt signaling, their role in regulating CK1α levels in CRC remains unclear. Regardless, reduced steady-state levels of CK1α render its overall activity rate limiting [8], providing increased selectivity for pharmacological activators of CK1α in neoplastic tissues (Figure 1).

## References

[1] Nusse R (2017). Cell.

[2] Lau T (2013). Cancer Res.

[3] Thorne CA (2010). Nat Chem Biol.

[4] Li B (2017). Sci Signal.

[5] Saraswati S (2010). PLoS One.

[6] Barham W (2013). Cancer Discov.

[7] Cruciat CM (2013). Science.

[8] Lebensohn AM (2016). Elife.

